# An edit script for taxonomic classifications

**DOI:** 10.1186/1471-2105-6-208

**Published:** 2005-08-25

**Authors:** Roderic DM Page, Gabriel Valiente

**Affiliations:** 1 DEEB, IBLS, University of Glasgow, Glasgow G12 8QQ, UK; 2 Department of Software, Technical University of Catalonia, E-08034 Barcelona, Spain

## Abstract

**Background:**

The NCBI taxonomy provides one of the most powerful ways to navigate sequence data bases but currently users are forced to formulate queries according to a single taxonomic classification. Given that there is not universal agreement on the classification of organisms, providing a single classification places constraints on the questions biologists can ask. However, maintaining multiple classifications is burdensome in the face of a constantly growing NCBI classification.

**Results:**

In this paper, we present a solution to the problem of generating modifications of the NCBI taxonomy, based on the computation of an edit script that summarises the differences between two classification trees. Our algorithms find the shortest possible edit script based on the identification of all shared subtrees, and only take time quasi linear in the size of the trees because classification trees have unique node labels.

**Conclusion:**

These algorithms have been recently implemented, and the software is freely available for download from .

## Background

The NCBI Taxonomy [1] provides one of the most powerful ways to navigate the National Center for Biotechnology Information (NCBI) sequence data bases. Every sequence in GenBank is associated with a taxon (which, however, may be unidentified), and each taxon has a unique place in the NCBI taxonomy. Hence, not only can the user retrieve sequences for a given species (such as *Homo sapiens*), but also for a group of species, such as mammals (Mammalia) or animals (Animalia).

The NCBI provides a single classification, assembled from a variety of sources including published literature, a panel of expert advisors, and the taxonomy provided by users when they submit new sequences. Given that there is not universal agreement on the classification of organisms, providing a single classification places constraints on the questions biologists can ask.

**Figure 1:**
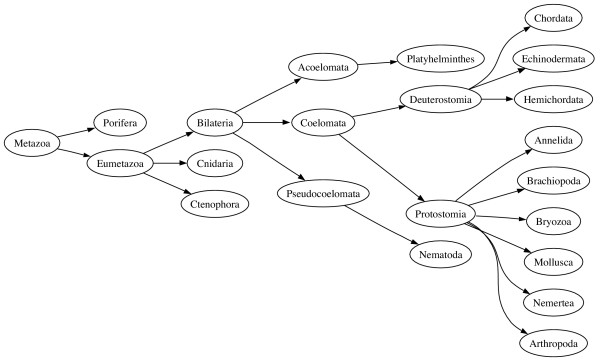
**Traditional view of animals**. A "traditional" view of animal relationships, based on the NCBI classification.

To give a concrete example, Figure 1 shows a simplified classification of animals, based on the current NCBI taxonomy. In this classification, the Bilateria are split into three groups (Acoelomata, Pseudocoelomata, and Coelomata) based on the nature of the internal body cavity (coelom). The Coelomata are themselves split into two groups, the Protostomia and the Deuterostomia, characterised by the fate of the blastopore during development (in the Protostomia this becomes the mouth, in the Deuterostomia it becomes the anus).

**Figure 2:**
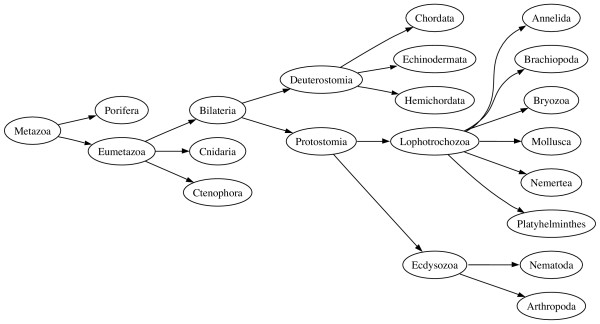
**An alternative view of animals**. A alternative tree of animals reflecting the "new animal classification".

An alternative view of animal classification is shown in Figure 2. The three-fold division based on body cavity disappears, leaving the fundamental split being between the Protostomia and the Deuterostomia. The Protostomia are divided into the Lophotrochozoa and the Ecdysozoa, the latter comprising arthropods, nematodes, and other moulting animals [2]. This classification has implications for comparative genomics. The best known animal genomes are *Homo sapiens *(human), *Drosophila melanogaster *(fly), and *Caenorhabditis elegans *(nematode). Under the classical classification (Fig. 1), the coelomates human and *Drosophila *are more closely related to either other than either is to the aceolomate *C. elegans*, suggesting it would be most productive to compare our genome with that of *Drosophila*, rather than the more distant nematode. However, in the alternative classification (Fig. 2) *Drosphila *and *C. elegans *are more closely related to each other than either is to humans, and we have no (phylogenetic) reason for choosing one over the other as a point of reference for interpreting the human genome. There is considerable debate about the merits of the two classifications [3-5]. However, because the NCBI provides only one classification users cannot, for example, easily query GenBank for all ecdysozoan sequences – this taxon simply does not exist in the NCBI database. Instead, users are forced to construct Boolean queries such as (Arthropoda AND Nematoda). While in this simplified example this is not a great hardship, as the trees get larger and the differences more profound, it becomes harder to pose a query that captures the taxa required.

One solution is simply to download the NCBI taxonomy, edit it to reflect the desired alternative classification, then use that to obtain sequences from taxa such as Ecdysozoa. It is reasonably straightforward to store a tree in a relational database an query it using SQL [6]. However, the NCBI taxonomy is continually growing as new organisms are sequenced. Hence, a locally edited classification will quickly become obsolete. Having to download a fresh copy and then manually edit it would quickly become tedious.

## Implementation

### Taxonomic classifications

Although ideally classifications mirror phylogenetic relationships, it is important to distinguish between classifications and phylogenies. A taxonomic classification can be modelled as a rooted, labelled, unordered tree. Unlike classifications, internal nodes of phylogenetic trees need not be labeled, although the internal nodes of a phylogeny may be decorated with measures of support (such as bootstrap values or Bayesian posterior probabilities).

### Subtree isomorphism

**Figure 3:**
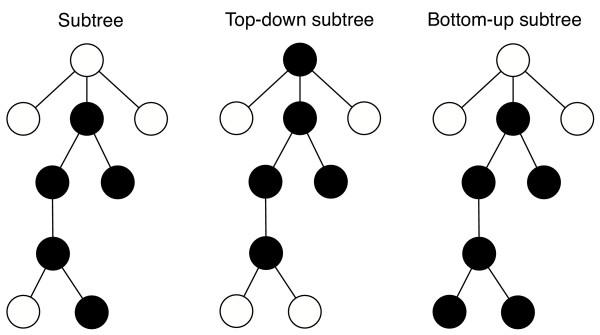
**Connected subgraph and top-down and bottom-up subtrees**. In the top-down subtree the parent of any node in the subtree is itself in the subtree. In the bottom-up matching, the children of any node in the matching are also in the matching. Modified from [10].

Our approach is to first find subtree isomorphisms between the two trees, *T*_1 _and *T*_2_. A subtree is a connected subgraph of a tree. We distinguish between *top-down *and *bottom-up *subtree isomorphism. A top-down node matching the parent of each node in the matching is itself in the matching (excluding the root which has no parent). In a bottom-up matching, all the children of a node in the matching are also in the matching (Fig. 3).

**Figure 4:**
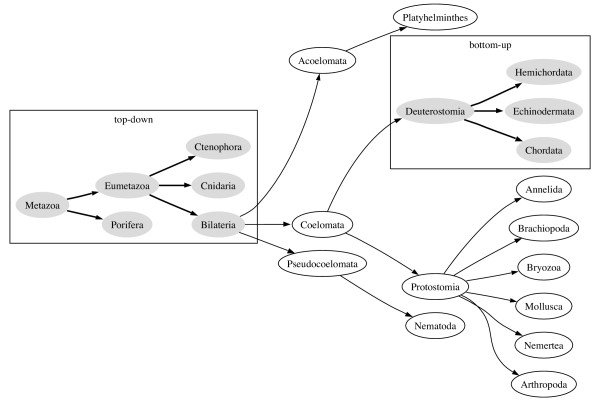
**Subtree isomorphisms**. The top-down and bottom-up subtree isomorphisms between the animal classifications shown in Figs. 1 and 2. (ignoring the trivial bottom-up subtrees that comprise a single leaf).

The algorithm first finds all subtrees, including bottom-up and top-down subtrees, that are common to *T*_1 _and *T*_2_. We find all kinds of subtree because, by themselves the subtrees found by each method can be small (Fig. 4).

### Script

Having identified common subtrees, we then list the operations needed to transform *T*_1 _into *T*_2_. The first step is to delete nodes in *T*_1 _that are not in any of the shared subtrees. The deletion of a node entails deletion of all the edges incident with the deleted node. We then add nodes found only in *T*_2_, and the corresponding edges. The size of the script depends on the size of the shared subtrees, hence it is desirable to find the largest such subtrees.

### Complexity

In general, computation of the least number of operations needed to transform *T*_1 _into *T*_2 _is an NP-hard problem [7], even for binary trees with a label alphabet of size two, as long as node and edge deletions, insertions, and label substitutions are allowed. However, in the case of trees with unique node labels, node label substitutions are forbidden because they may generate trees with non-unique node labels [8], and the least number of operations or edit distance becomes a function of the size of shared subtrees [9]. By identifying the largest common subtrees, we obtain the shortest possible edit script.

### Computing an edit script

Taxonomic classifications are modelled as trees with unique node labels, and this fact makes it easier to deal with trees in terms of their sets of node labels and node label pairs, as done for graphs with unique node labels in [8].

**Definition 1 ***Let T *= (*V,E*) *be a tree*. *The label representation of T, denoted by R(T), is given by R*(*T*) = (*L,C*), *where L *= {ℓ(*v*) | *v *∈ *V*} *and C *= {(ℓ(*v*),ℓ(*w*)) | (*v,w*) ∈ *E*}.

Thus, the label representation *R*(*T*) of a tree *T *defines the equivalence class of all those trees that are isomorphic to *T*. The use of label representations simplifies the notation, because isomorphic trees have exactly the same label representation.

The edit operations of node and edge deletion and insertion, allow one to transform any given tree into any other tree. Label substitutions are forbidden because they may generate trees with non-unique node labels [8].

**Definition 2 ***Let T*_1 _= (*V*_1_,*E*_1_) *and T*_2 _= (*V*_2_,*E*_2_) *be trees, let R*(*T*_1_) = (*L*_1_, *C*_1_), *and let R*(*T*_2_) = (*L*_2_,*C*_2_). *Let also C *= *L*_1 _∪ *L*_2 _∪ {λ}.

*A node edit operation between T*_1 _*and T*_2 _*is a pair *(*a*, *b*) ∈ *C *× *C *with *a *≠ λ *or b *≠ λ. *A node edit operation of the form *(*a*, λ) *establishes deletion of the node v *∈ *V*_1 _*with *ℓ(*v*) = *a together with the edge *(*parent*(*v*), *v*), *if v is not the root of T*_1_, *and deletion of edge *(*v*,*x*) *for each child x of v in T*_1_. *A node edit operation of the form *(λ,*b*) *establishes insertion of the node w *∈ *V*_2 _*with *ℓ(*w*) = *b*.

*An edge edit operation between T*_1 _*and T*_2 _*is a triple *(*a*, *b*, *c*) ∈ *C *× *C *× *C with b *≠ λ *and a *≠ λ *or c *≠ λ. *An edge edit operation of the form *(*a*, *b*, λ) *establishes deletion of the edge *(*v*, *x*) ∈ *E*_1 _*with *ℓ(*v*) = *a and *ℓ(*x*) = *b*, *and an edge edit operation of the form *(λ, *b*, *c*) *establishes insertion of the edge *(*w*,*y*) ∈ *E*_2 _*with *ℓ(*w*) = *b and *ℓ(*y*) = *c*.

An edit operation is either a node edit operation or an edge edit operation.

An edit script between two trees is just a set of edit operations that, if applied in the right order (essentially, inserting an edge only after having inserted the nodes incident with the inserted edge), allow one to transform one tree into the other.

**Definition 3 ***An edit script between two trees T*_1 _= (*V*_1_,*E*_1_) *and T*_2 _= (*V*_2_,*E*_2_) *is a set of edit operations that transform R*(*T*_1_) *into R*(*T*_2_).

Given *R*(*T*_1_) = (*L*_1_, *C*_1_) and *R*(*T*_2_) = (*L*_2_, *C*_2_), an edit script between *T*_1 _and *T*_2 _can be easily obtained by sorting the label sets and computing set differences, as follows:

• Delete all nodes with labels in *L*_1 _\ *L*_2_

• Insert all nodes with labels in *L*_2 _\ *L*_1_

• Delete all edges with labels in *C*_1 _\ *C*_2_

• Insert all edges with labels in *C*_2 _\ *C*_1_

However, such a procedure does not, in general, lead to the shortest possible edit script, because some of the edge deletion operations may be redundant, given that deletion of a node entails deletion of all the edges incident with the deleted node. While any edit script would suffice to transform one tree into the other, the shortest edit script leads to a faster computation of the edited tree, given the script and the original tree.

The following, alternative procedure is based on the set of common node labels between the two trees, which can be easily obtained as the intersection of the sets of node labels in the label representation of the trees, that is, *C *= *L*_1 _∩ *L*_2 _= {ℓ(*v*) | *v *∈ *V*_1_} ∩ {ℓ(*w*) | *w *∈ *V*_2_}. The procedure can be sketched as follows:

• Delete all nodes *v *∈ *V*_1 _with ℓ(*v*) ∉ *C*.

• Insert all nodes *w *∈ *V*_2 _with ℓ(*w*) ∉ *C*.

• Delete all edges (*v*,*x*) ∈ *E*_1 _with ℓ(*v*), ℓ(*x*) ∈ *C *and such that the node *w *∈ *V*_2 _with ℓ(*v*) = ℓ(*w*) is not the parent in *T*_2 _of the node *y *∈ *V*_2 _with ℓ(*x*) = ℓ(*y*).

• Insert all edges (*w*,*y*) ∈ *E*_2 _with ℓ(*w*), ℓ(*y*) ∈ *C *and such that the node *v *∈ *V*_1 _with ℓ(*v*) = ℓ(*w*) is not the parent in *T*_1 _of the node *x *∈ *V*_1 _with ℓ(*x*) = ℓ(*y*).

• Insert all edges (*w*, *y*) ∈ *E*_2 _such that ℓ(*w*) ∉ *C *or ℓ(*y*) ∉ *C*.

**Figure 5:**
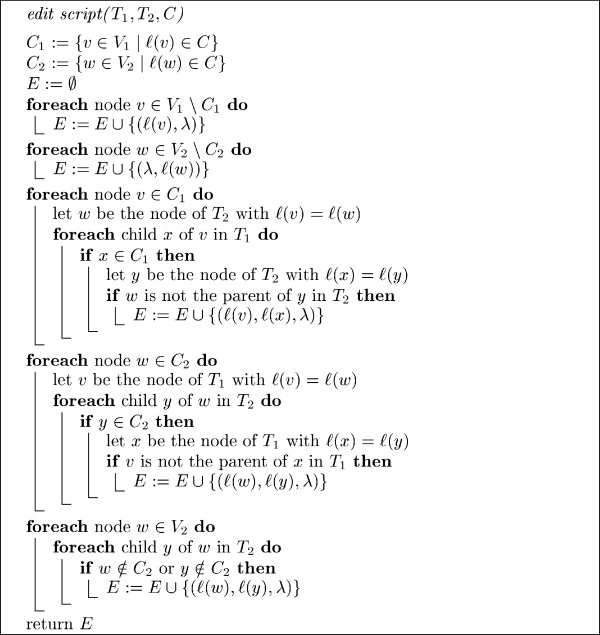
**Algorithm for computing edit script**. Let *C *be a set of common node labels of *T*_1 _and *T*_2_. A function call of the form *edit script *(*T*_1_, *T*_2_, *C*) returns a set *E *of elementary edit operations that transform *T*_1 _into *T*_2_.

A detailed description of the algorithm is given in Fig. 5. Correctness of the edit script algorithm is easy to establish.

**Theorem 1 ***Let T*_1 _*and T*_2 _*be trees, let C *⊆ ℓ(*V*_1_) ⋂ ℓ(*V*_2_), *let E *= *edit script *(*T*_1_, *T*_2_, *C*), *and let **be the result of applying the set of edit operations in E to T*_1_. *Then*, *and T*_2 _*are isomorphic*.

**Proof **It has to be shown that . Let *R*(*T*_1_) = (*L*_1_, *C*_1_) and *R*(*T*_2_) = (*L*_2_, *C*_2_). The edit script establishes the deletion of all nodes with labels in *L*_1_\ *C *and the insertion of all nodes with labels in *L*_2 _\ *C*. Thus,  = *L*_1 _\ (*L*_1 _\ *C*) ∪ (*L*_2 _\ *C*) = *C *∪ (*L*_2 _\ *C*) = *L*_2_.

**Figure 6:**
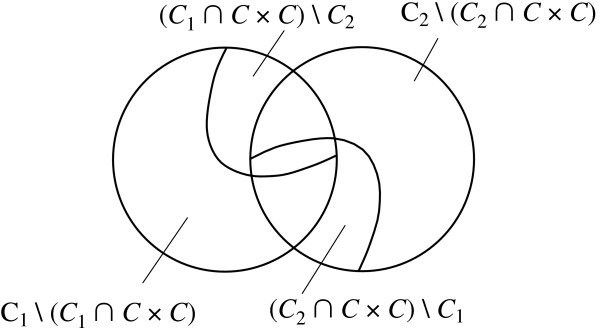
**Illustration for the proof of Theorem 1**. Given the label representation *R*(*T*_1_) = (*L*_1_, *C*_1_) and *R*(*T*_2_) = (*L*_2_, *C*_2_) of two trees, and a set of common node labels *C *⊂ *L*_1 _∩ *L*_2_, *T*_1 _can be transformed into *T*_2 _by deleting all nodes with labels in *L*_1 _\ (*C*, which implies deletion of all edges with source and target node labels in *C*_1 _\ (*C*_1 _∩ *C *× *C*); inserting all nodes with labels in *L*_2 _\ *C*, deleting all edges with source and target node labels in (*C*_1 _∩ *C *× *C*) \ *C*_2_; inserting all edges with source and target node labels in (*C*_2 _∩ *C *× *C*) \ *C*_1_; and inserting all edges with source and target node labels in *C*_2 _\ (*C*_2 _∩ *C *× *C*).

The edit script also establishes the deletion of all edges with source and target labels in (*C*_1 _∩ *C *× *C*) \ *C*_2_, the insertion of all edges with source and target labels in (*C*_2 _∩ *C *× *C*) \ *C*_1_, and the insertion of all edges with source or target label in *L*_2 _\ *C*, that is, of all edges in *C*_2 _\ (*C*_2 _∩ *C *× *C*). Furthermore, the deletion of all nodes with labels in *L*_1 _\ *C *entails the deletion of all edges with source or target label in *L*_1 _\ *C*, that is, of all edges in *C*_1 _\ (*C*_1 _∩ *C *× *C*). (See Fig. 6.)

Now, *C*_1 _= (*C*_1 _\ *C*_2_) ∪ (*C*_1 _∩ *C*_2_) = ((*C*_1 _∩ *C *× *C*) \ *C*_2_) ∪ ((*C*_1 _\ (*C*_1 _∩ *C *× *C*)) \ *C*_2_) ∪ (*C*_1 _∩ *C*_2_). In a similar vein, *C*_2 _= ((*C*_2 _∩ *C *× *C*) \ *C*_1_) ∪ ((*C*_2 _\ (*C*_2 _∩ *C *× *C*)) \ *C*_1_) ∪ (*C*_1 _∩ *C*_2_).

Thus,

 = *C*_1 _\ ((*C*_1 _∩ *C *× *C*) \ *C*_2_) \ ((*C*_1 _\ (*C*_1 _∩ *C *× *C*)) \ *C*_2_) ∪ ((*C*_2 _∩ *C *× *C*) \ *C*_1_) ∪ ((*C*_2 _\ (*C*_2 _∩ *C *× *C*)) \ *C*_1_) = (*C*_1 _∩ *C*_2_) ∪ ((*C*_2 _∩ *C *× *C*) \ *C*_1_) ∪ ((*C*_2 _\ (*C*_2 _∩ *C *× *C*)) \ *C*_1_) = *C*_2 _and therefore,  = (*L*_2_, *C*_2_) = *R*(*T*_2_), that is,  and *T*_2 _are isomorphic.

The edit script algorithm can be implemented to take time quasi linear in the size of the trees, by using any efficient dictionary data structure to represent the set of common node labels. The same dictionary data structure allows one to compute the set of common node labels within the same time bound and thus, the whole procedure can be implemented to take time quasi linear in the size of the trees. In particular, our C++ implementation uses the STL associative container set<string> as representation of the set of shared node labels.

## Results

Here we suggest a solution based on the notion of an "edit script" that summarises the differences between two trees. Given two trees, *T*_1 _and *T*_2_, a script lists the operations required to convert *T*_1 _into *T*_2_. The script could be constructed manually, but it would be more efficient to generate it automatically. Hence, we imagine the following scenario. A user downloads the NCBI taxonomy tree (or that subtree relevant to their interests), then edits the tree to reflect their preferred classification. Using the algorithm we describe below, the user then computes the edit script that transforms the NCBI tree into their classification. When a new NCBI tree appears on the NCBI ftp site, the user downloads that tree and applies to edit script to regenerate their classification. In this way, the user need only edit the NCBI tree once.

As an example, given the two trees in Figs. 1 and 2, the edit script for these trees is:

delete node Pseudocoelemata

delete node Coelomata

delete node Protostomia

delete node Acoelomata

insert node Ecdysozoa

insert node Lophotrochozoa

insert node Protostomia

insert edge Bilateria -> Deuterostomia

insert edge Bilateria -> Protostomia

insert edge Ecdysozoa -> Nematoda

insert edge Ecdysozoa -> Arthropoda

insert edge Lophotrochozoa -> Annelida

insert edge Lophotrochozoa -> Brachiopoda

insert edge Lophotrochozoa -> Bryozoa

insert edge Lophotrochozoa -> Mollusca

insert edge Lophotrochozoa -> Nemertea

insert edge Lophotrochozoa -> Platyhelminthes

insert edge Protostomia -> Lophotrochozoa

insert edge Protostomia -> Ecdysozoa

**Figure 7:**
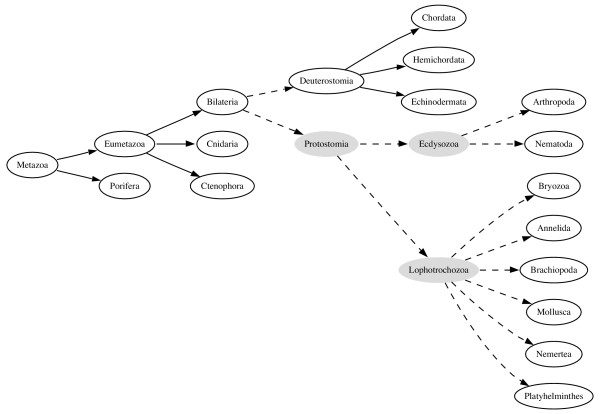
**Result of applying the edit script**. The result of applying the edit script to the tree in Fig. 1. This tree is the same as that shown in Fig. 2. Nodes which have been inserted into the tree are filled with light grey. A dashed line represents an edge that has been added to the original tree.

Applying the script to the NCBI tree (Fig. 1) yields the tree shown in Fig. 7, which is identical to the tree shown in Fig. 2.

## Discussion

The size of the edit script will be a function of the size of the input trees, and the degree to which they differ. At the time of writing, there are 83,802 metazoan taxa in GenBank. Given that the disagreement between the Coelomata and Ecdysozoa hypotheses concerns the deep level relationships, we can simplify the task by reducing the subtrees about which there is little or no disagreement to single nodes. For example, the 36,746 arthropod taxa can be represented by a single node. Hence, the tree shown in Fig. 1 is greatly simplified, compared to the complete NCBI tree.

One issue we don't directly address here is using the tree that results from applying the edit script to query GenBank. There are at least two approaches to doing this. The first is to store the tree in a local database and use a method such as visitation numbers [6] to generate queries involving higher taxa (such as listing all sequences from the Ecdysozoa).

Another approach would be to use the tree to rewrite queries in terms of the original GenBank taxonomy. For example, in our rather simplified example in Fig. 2, we could use the tree to automatically rewrite the query term "Ecdysozoa" as the sum of its children (Arthropoda and Nematoda) as both trees (Fig. 1 and Fig. 2) agree on the composition of these two taxa. One advantage of this approach is that we can continue to use tools such as BLAST, but in the context of a different taxonomic classification.

## Conclusion

We present a solution to the problem of generating modifications of the NCBI taxonomy, based on the computation of an edit script that summarises the differences between two classification trees. Our algorithms find the shortest possible edit script based on the identification of all shared subtrees, and only take time quasi linear in the size of the trees because classification trees have unique node labels. We have implemented the edit function in a C++ program that makes use of the Graph Template Library (GTL) available from . The code has been compiled and tested with the GNU gcc compiler on Mac OS X and Linux machines, and is available from . The software comprises two programs, forest and script. The program forest takes two trees in GML format (the original tree and the edited tree) and computes an edit script. Given this script and the original tree, script generates the edited tree.

## Availability and requirements

• **Project name: **Forest

• **Project home page: **

• **Operating system(s): **Unix/Linux, tested on Mac OS X and Red Hat 8.0

• **Programming language: **e.g. C++

• **Other requirements: **Graph Template Library (GTL) ()

• **License: **GNU GPL

• **Any restrictions to use by non-academics: **Forest depends on GTL, which can be downloaded free of charge for non-commercial use. Commercial use of GTL requires a licence from BRAINSYS – Informatiksysteme GmbH ()

## Authors' contributions

RDMP posed the problem, and GV developed the algorithm. RDMP and GV jointly developed the software and wrote the manuscript.
